# Correction: An event related potential study of inhibitory and attentional control in Williams syndrome adults

**DOI:** 10.1371/journal.pone.0177587

**Published:** 2017-05-08

**Authors:** The PLOS ONE Staff

## Notice of Republication

This article was republished on April 27, 2017 to correct an error in the title introduced during the typesetting process. The publisher apologizes for this error. Please download this article again to view the correct version. The originally published, uncorrected article and the republished, corrected article are provided here for reference.

## Supporting information

S1 File Originally published, uncorrected article. (PDF)

S2 File Republished, corrected article. (PDF)
