# Supplementary material for: Correction: An event related potential study of inhibitory and attentional control in Williams syndrome adults
Source: PLoS One. 2017 May 8;12(5):e0177587. doi: 10.1371/journal.pone.0177587 (PMC5421807; doi:10.1371/journal.pone.0177587)
Supplement: S1 File — (PDF) [file pone.0177587.s001.pdf]

RESEARCH ARTICLE

An event related potential study of ihibitory and attentional control in Williams syndrome adults

Joanna M. H. Greer[1], Colin Hamilton[1], Mhairi E. G. McMullon[1☯], Deborah M. Riby[2☯], Leigh M. Riby[1]*

1 Department of Psychology, Northumbria University, Newcastle-upon-Tyne, United Kingdom, **2** Department of Psychology, Durham University, Durham, United Kingdom

☯ These authors contributed equally to this work.
* leigh.riby@northumbria.ac.uk

Check for updates

OPEN ACCESS

Citation: Greer JMH, Hamilton C, McMullon MEG, Riby DM, Riby LM (2017) An event related potential study of ihibitory and attentional control in Williams syndrome adults. PLoS ONE 12(2): e0170180. doi:10.1371/journal.pone.0170180

Editor: Alexandra Key, Vanderbilt University, UNITED STATES

Received: May 30, 2016

Accepted: December 30, 2016

Published: February 10, 2017

Copyright: © 2017 Greer et al. This is an open access article distributed under the terms of the Creative Commons Attribution License, which permits unrestricted use, distribution, and reproduction in any medium, provided the original author and source are credited.

Data Availability Statement: The data are available at the OSF repository. DOI 10.17605/OSF.IO/328A8 | ARK c7605/osf.io/328a8.

Funding: The research was conducted as part of a PhD thesis, therefore there was no source of external or internal funding apart from the student's travel expenses supplied by the Psychology department at Northumbria University.

Competing interests: The authors have declared that no competing interests exist.

Abstract

The primary aim of the current study was to employ event-related potentials (ERPs) methodology to disentangle the mechanisms related to inhibitory control in older adults with Williams syndrome (WS). Eleven older adults with WS (mean age 42), 16 typically developing adults (mean age 42) and 13 typically developing children (mean age 12) participated in the study. ERPs were recorded during a three-stimulus visual oddball task, during which participants were required to make a response to a rare target stimulus embedded in a train of frequent non-target stimuli. A task-irrelevant infrequent stimulus was also present at randomised intervals during the session. The P3a latency data response related to task-irrelevant stimulus processing was delayed in WS. In addition, the early perceptual N2 amplitude was attenuated. These data are indicative of compromised early monitoring of perceptual input, accompanied by appropriate orientation of responses to task-irrelevant stimuli. However, the P3a delay suggests inefficient evaluation of the task-irrelevant stimuli. These data are discussed in terms of deficits in the disengagement of attentional processes, and the regulation of monitoring processes required for successful inhibition.

Introduction

Williams syndrome (WS) is a neurodevelopmental disorder with an estimated prevalence of 1:20,000 [1], caused by a micro-deletion of approximately 28 genes on chromosome 7 (7q11.23) [2]. Behavioural and cognitive outcomes have been linked to a number of candidate genes associated with neuronal development and expression (e.g. LIMK1, CYLN2, GTF21; see [3] for a review). Although there is significant heterogeneity of cognitive function [4], individuals with WS tend to function at the level of mild-to-moderate intellectual difficulty [5]. The disorder has attracted the attention of cognitive scientists primarily due to the distinctive cognitive profile. Indeed, an abundance of literature has documented relatively more impaired visuo-spatial skills (e.g. [6, 7]) compared with relatively less impaired verbal processing [8], though always against a background of mild-moderate intellectual difficulty. Although the

heterogeneity of cognitive functioning is mirrored in the vast behavioural variability seen in the disorder [9], many individuals with WS (both children and adults) tend to be highly-sociable, exhibiting a strong desire to converse with others, and an eagerness to make eye contact with, and to indiscriminately approach strangers [10, 11].

Many facets of the behavioural and social phenotype in WS, such as social disinhibition [12, 13], their lack of stranger danger awareness [14, 15], and propensity for prolonged face-gazing [16] are associated with atypicalities in frontally controlled executive function (EF) processes. Due to the heterogeneity of executive processing mechanisms subserved by the frontal lobes, there are discrepancies in the literature with regard to the EF functions affected in WS [17, 18], and vast individual differences are evident. However, research reporting deficits in inhibition [19–22], visual and auditory sustained attention [19, 22, 23], visual selective attention [24], and attentional set-shifting [25] prove promising in elucidating specific executive processes impaired and how these may explain the behavioural and social characteristics associated with the syndrome.

Research which adopts a *Go / No-Go* paradigm is particularly informative when examining attentional and inhibitory profiles in both typical developing individuals and those with developmental disorders such as WS. In a typical *Go / No-Go* task, participants are required to make a motor response (*Go*) to a frequently presented stimulus and withhold a response (*No-Go*) to an infrequent target stimulus. During the task, participants become habituated to the frequent stimuli and relatively automatic responding begins to occur. Consequently, withholding a response to *No-Go* trials becomes problematic. Individual differences in the measurement of withholding a response to the target stimuli have been shown to be related to inhibitory ability and impaired frontal lobe function (see [26] for a meta-analysis). A recent study employing the Sustained Attention Response Task (SART, a computerised *Go / No-Go* paradigm) is particularly informative here [22]. Greer and colleagues [22] considered multiple measures of executive control and inhibition in adults with WS (e.g. reaction time (RT) after an error, false alarms (FAs), overall RT variability) and concluded such indices as having great value in evaluating everyday cognitive challenges. Increased FAs to the infrequent target stimulus were indicative of impaired functioning of frontal brain regions sub-serving inhibitory control, as previously reported in the syndrome ([27]; see also the Attention-Deficit Hyperactivity Disorder (ADHD) / WS work on inhibition [28]). Interestingly, and much like other populations with frontal executive control deficits such as traumatic brain injury (TBI) [29], post error slowing was also compromised in the WS group. This failure to re-engage attention after an error has elsewhere been linked with impaired cognitive abilities and spatial cognition deficits in WS [30]. Overall, Greer et al. [22] promote the use of *Go / No-Go* paradigms to disentangle the numerous executive processes related to inhibition and attentional control which are proposed to be problematic in the disorder.

Converging evidence from studies adopting *Go / No-Go* paradigms and neuroimaging techniques such as functional magnetic resonance imaging (fMRI) and event—related potentials (ERP) methodologies have enabled researchers to identify the spatial / functional mapping and temporal dynamics of fronto-cortical networks recruited during attentional and inhibitory processes in both typically and atypically developing individuals. Particularly relevant to the current investigation, Mobbs and colleagues [27] compared the fMRI profile of individuals with WS and typically developing individuals matched for chronological age and gender. Despite comparable behavioural performance (accuracy but not RT) between groups, compared to the typical controls the WS group reported dis-engagement of the frontal-striatal networks of the brain which contribute to the complex pattern of social and behavioural deficits associated with WS [14, 31], and also increased activity in the posterior cingulate cortex on presentation of *No-Go* trials. This demonstrates that, irrespective of behavioural similarities,

these individuals with WS reported a) hypoactivity in the fronto-cortical and subcortical structures associated with behavioural inhibition, and b) hyperactivity in posterior regions which, in ADHD, has been linked with a reduced ability to reallocate attention after an error [32] and which was a main finding of Greer et al. [22]. Research employing ERP methodology in WS is scarce; however there is evidence of atypical neural activity in frontal regions in response to social stimuli (faces; [33]) and which may be linked with the social disinhibition associated with the syndrome (e.g. [14]). In contrast, atypically enhanced frontal ERP activity in response to non-social stimuli (houses) compared with social stimuli (faces) is reported, and contrary to the pattern hypothesised [34]. The dearth of research focusing on the ERP correlates which sub-serves the behavioural and cognitive profile of individuals with WS makes interpretation of these conflicting findings more challenging.

Here we aim to contribute to the theoretical understanding of the adult WS cognitive profile by examining attentional and inhibitory control mechanisms in the disorder using the temporal precision of ERP methodology and a three-stimulus Oddball paradigm (Oddball; [35]). In contrast to the two-stimulus *Go / No-Go* methodology described above, the Oddball paradigm requires participants to respond to an infrequent target stimulus while withholding their response to two distractors; a frequent non-target stimulus and an infrequent task-irrelevant novel stimulus. It measures automatic shifts in attention to task-irrelevant information, the allocation of cognitive resources to task-relevant stimuli, and has been proposed to be associated with context updating in working memory (see [36] for a review). Notably, the ERP response to the task-irrelevant novel stimulus is thought to reflect the processes engaged in order to successfully inhibit task-irrelevant information. Topographical distributions to infrequent task-irrelevant novel stimuli can be observed over bilateral frontal and superior temporal regions, and which have been related to the inhibition of motor responses in a cognitive task [37]. Thus, the inclusion of an infrequent task-irrelevant novel stimulus in the current Oddball task enables us to observe the cortico-electrical activity evoked when unexpected behavioural inhibitory control is required [38]. Of note, whilst it is important to dichotomise between one infrequent task-irrelevant 'deviant' stimulus and multiple unrepeated infrequent task-irrelevant 'novel' stimuli (see [39] for a discussion), both require the inhibition of motor action. We have previously demonstrated that a single infrequent task-irrelevant stimulus repeated throughout the task elicits the fronto-central distribution expected during successful response inhibition in typically developing adults [40], thus we will refer to the infrequent task-irrelevant stimulus as 'novel' here, in order to simplify description of the task and results.

Three main ERP components are elicited during the completion of the task, the N2, P3a and P3b. The N2 is a negative going waveform which peaks between ~180–350 ms post stimulus, and is associated with the early recognition and parsing of visual information in the environment [41]. Daffner and colleagues [42] have been influential in characterising the functional significance of the N2 ERP component. For instance, the N2 evoked when no behavioural response is required (i.e. novel stimulus), typically reports a fronto-central scalp distribution and is elicited typically without conscious awareness. In contrast, the N2 evoked in response to the target stimulus represents the degree of attention that is needed for processing stimuli context and is typically observed centro-parietally (see [43] for a detailed review on the classification and function of the N2 component). Elsewhere, the importance of top-down processes and visual selective attention in the generation of the N2 has been emphasised [44]. The P3a and P3b are subcomponents of the positive going P300 waveform, and have different functional correlates [36]. The P300 typically peaks between ~250–500ms post-stimulus, with the P3a reporting a fronto-central distribution, and the P3b a centro-parietal distribution [45]. The P3a is associated with automatic responses during the engagement of attention, inhibition, and orienting resources to items in the environment. As such, it typically presents relatively

larger frontal peak amplitude and relatively short peak latency duration. The P3a has also been associated with dopaminergic function and attentional control processes [46, 47]. The P3b is associated with the controlled processes required during working memory storage updating, and relative to the P3a, typically reports a smaller peak amplitude and later peak latency [48], reflecting the greater amounts of attentional resources required for task performance (see [36] for a detailed review of the classification and function of the P300 component).

The Oddball paradigm has been used widely in research investigating neural functioning of TD individuals [40], clinical and subclinical populations (e.g. schizophrenia [49], eating disorders [50], and developmental disorders (e.g. ASD: [51]). To date, the Oddball task as described here has not been employed in research with individuals with WS, though there is evidence for atypical activity in WS in components elicited by the Oddball task [33, 52, 53]. However, one known study is informative as to the profile of the P3a and P3b that may be observed in WS during an Oddball task. Key and Dykens [54] employed an Oddball-type paradigm to investigate global / local stimulus discrimination during a Navon style visuo-spatial task in a group of adults with WS and CA controls. Relative to a standard stimulus, the WS group reported shorter P3a latency and greater P3a amplitude in response to the global stimulus, but no difference in P3a amplitude or latency in response to the local stimulus, suggesting insufficient allocation of attentional resources to local features. In contrast, while the CA group reported increased P3b latencies in response to the local targets, the WS group reported no P3b discrimination between conditions, indicative of impaired effortful processing when greater attentional resources are required, as would be the case during local stimulus discrimination.

The aim of the current study is to characterise the neural signature of adults with WS during a visual three-stimulus Oddball task, and thus elucidate the neural mechanisms that may underpin the deficient attentional and inhibitory profiles associated with the syndrome. Two comparison groups are included in the study; a cohort of typically developing adults matched for chronological age (CA), and a group of typically developing children matched for verbal mental ability (MA). Typically developing younger children display an age-associated ERP profile which reflects their ongoing neuronal maturational processes [55, 56]. Thus, we do not predict an ERP profile in adults with WS that is indicative of verbal mental age; however the MA group are included in the study for completeness. Based on the previous ERP research with WS [54], ADHD [57], autism spectrum disorder (ASD) [58], and recent behavioural findings [22], we predict a profile indicative of atypical attentional and inhibitory processing. However, due to the novelty of the study and because we cannot be sure how the deficits will manifest we ask a number of questions. Compared to the CA group will adults with WS demonstrate: 1) atypical earlier attentional processing indexed by attenuated N2 peak amplitude and / or latency differences in response to the task-irrelevant novel and target stimuli? 2) increased P3a latency reflecting a delay in the orienting to novelty response and or amplitude difference, and 3) increased P3b latency and amplitude difference indicative of working memory and storage updating functioning.

Method

Participants

Three groups participated; adults with Williams Syndrome (WS), and two comparison groups (see [59] for a discussion of matching procedures) consisting of a group of typically developing adults matched for chronologically age and gender (CA), and typically developing children matched for verbal mental ability (MA). Eleven adults with WS (7 males, aged 37yrs 2mths - 49yrs 3mths, mean age 42yrs 7mths, SD 48mths) were recruited via the Williams Syndrome Foundation. Nine had their genetic diagnosis confirmed with fluorescence in situ hybridisation

(*FISH*) testing, whilst the remainder had been diagnosed based on their clinical phenotype prior to the availability of genetic diagnosis. Seven of the WS group lived at home with their parents / or with carers in sheltered accommodation, and four lived independently. Six were in some form of paid employment / volunteer work while the rest attended daycare centres or receive state-proved care assistance.

The CA group consisted of sixteen typically developing adults (9 males, aged 36yrs 10mths - 49yrs 2mths, mean age 42yrs 10mths, SD 50mths) matched for chronological age. The MA group comprised of thirteen typically developing children (7 males, aged 8yrs 7mths -15yrs 7mths, mean age 11yrs 2mths, SD 25mths) and who were matched to the WS group for receptive vocabulary using the raw scores from the British Picture Vocabulary Scale (BPVS-II) [60]. Mean raw BPVS scores were WS 116.82 *(SD 10.36)*, MA 115.8 *(SD 14.16)* ($t(22) = 1.148$, $p = .884$). Any participants in both the CA and MA groups reporting a developmental disorder diagnosis (e.g. ADHD and ASD) were excluded from the study. Written informed consent was provided by all participants in the WS, CA, and, MA groups, and by parents / carers of both the WS and MA groups.

Ten of the WS group (7 males, mean age 41yrs 6mths, SD 39mths), thirteen of the CA-matched adults (4 males, mean age 42yrs 3mths, SD 51mths), and twelve of the MA-matched children (6 males, mean age 11yrs 3mths, SD 25mths) were included in the final analysis. Data from one WS participant, three CA participants and one MA participant were excluded due to high levels of EEG artefacts which compromised further analysis.

Handedness from all participants was assessed using the Edinburgh Handedness Inventory (EHI) [61]. Four of the WS group were left-handed, while all participants in the CA and MA groups were right-handed. The participants in the two comparison groups received £6.00 for their participation. This study received ethical clearance from the local ethics committee.

Materials and procedure

The three-stimulus Oddball task was programmed and presented using E-Prime presentation software on a Toshiba laptop with 14in. monitor. The task comprises of frequent, novel, and target stimuli. The target stimulus (red circle, area = 12.6 cm^2) appeared on 13% of trials, the standard frequent stimulus (green square, area = 16 cm^2) appeared on 74% of trials, and the novel stimulus (blue square, area = 256 cm^2) appeared on 13% of trials. Participants completed a 10-trial practice block. The testing phase consisted of 2 blocks of 150 trials each. Stimuli remained on screen for 250ms, and were followed by an inter-stimulus interval, randomised between 830ms and 930ms. Participants were instructed to press the space bar in response to the target stimulus and ignore all other stimuli (see [45] for further discussion of the Oddball task and in particular stimulus parameters that affect the generation of the ERP components). The nature of the Oddball task mimicked previous research which has successfully generated the ERP components of interest [40, 50].

The testing sessions with the WS group took place in their homes in a quiet room with electrical noise conditions controlled to mimic laboratory conditions [62, 63]. A parent / carer was either present at the session or nearby. The comparison groups' testing sessions took place in the Psychology Department at the host University or in the participants own homes, with the same control for electrical noise as previously described. The experimenter outlined the experimental procedure, and invited each participant to read and sign an informed consent form and complete the EHI.

EEG recording

The EEG was recorded from 32 channels using an electrode cap (Biosemi, Amsterdam, The Netherlands). Electrode placement was based on the extended international 10–20 system [64]. The montage included 4 midline sites (FZ, CZ, PZ, OZ), 14 sites over the left hemisphere (Fp1, AF3, F3, F7, Fc1, Fc5, C3, T7, Cp1, Cp5, P3, P7, Po3, O1), and 14 sites over the right hemisphere (Fp2, Af4, F4, F8, Fc2, Fc6, C4, T8, Cp2, Cp6, P4, P8, Po4, O2). Additional electrodes were placed on the left and right mastoids for referencing purposes. Electrodes were placed above and below the left eye to record the vertical electrooculogram to assess eye blink movement. Horizontal eye movements were removed manually during ERP processing.

ERP processing

All signals were digitized at a rate of 2048 Hz, with a recording epoch of 1,000ms (-200 to +800ms). High-pass filter settings were 0.05 – 45Hz, and baseline corrected to -200 μV. Automatic eye blink correction, artefact rejection (values outside the range of −100 μV to +100 μV), and ERP averaging were carried out off-line using Neuroscan SCAN 4.5 software (Compumedics, El Paso, TX). After eye blink correction and removal of trials with artefacts, the remaining trials were used in the analysis of each group's responses, with a minimum of sixteen trials per condition / participant required for inclusion in the final data analysis. There were no differences in the trials contributing to the ERPs for the standard (WS = 149, MA = 162, CA = 120; $p>0.05$), target (WS = 27, MA = 30, CA = 25; $p>0.05$) and novel stimuli (WS = 28, MA = 27, CA = 23; $p>0.05$). The components of interest were N2, P3a, and P3b, detected in the time frames 200-325ms, 310-450ms, and 380-600ms respectively, based on visual inspection of the individual waveforms, employing the automatic peak detection procedures in Neuroscan in the aforementioned time windows. Employing a targeted approach, these data were obtained from the midline sites (FZ, CZ, and PZ) and where peaks were maximal, based on visual inspection of the grand average ERPs and previous research employing the Oddball task [40, 65–67].

Data analysis

The peak amplitude and latencies for the ERP components of interest were investigated, with all analyses conducted using SPSS version 21. The between-subjects factors were group (WS, CA, MA), and the within-subjects factors were electrode site (FZ, CZ, PZ). Cohens *d* (bias corrected; [68]) are reported for WS group differences as a measure of effect size.

Results

ERP data were analysed with a 3 x 3 analysis of variance (ANOVA), with group (WS, CA, MA) as the between measures factor, and site (FZ, CZ, PZ) as the within measures factor. Follow-up / planned comparisons of group and site differences were investigated using t-tests. Results upheld Mauchly's test of sphericity unless stated. Where this test was violated, a Greenhouse-Geisser correction was applied to the results. ERP waveforms in response to the novel and target stimuli are presented in Figs 1 and 2.

N2 results

N2 novel. The N2 (novel) amplitude and latency data were calculated from the mean of the raw peak amplitude and latency scores in response to the novel stimulus. Descriptive statistics for peak N2 amplitude and peak N2 latency to the novel stimuli are presented in Table 1.

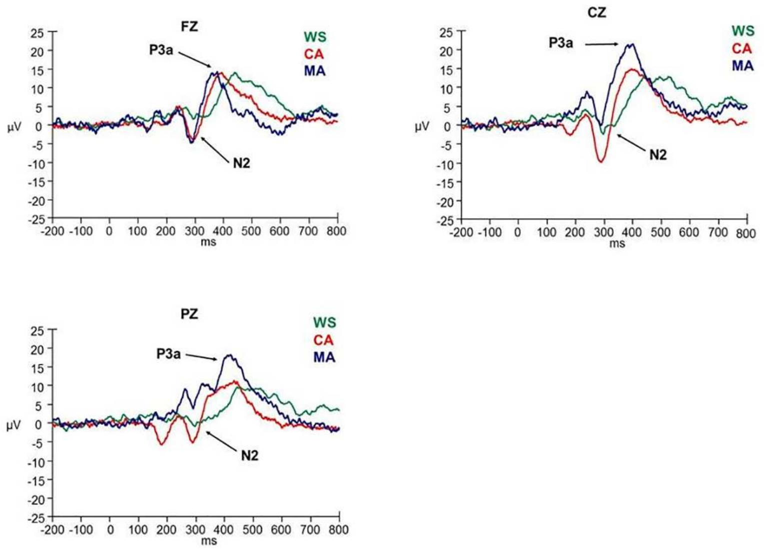

Fig 1. ERP waveforms in response to the novel stimulus at FZ, CZ, and PZ electrode sites.

doi:10.1371/journal.pone.0170180.g001

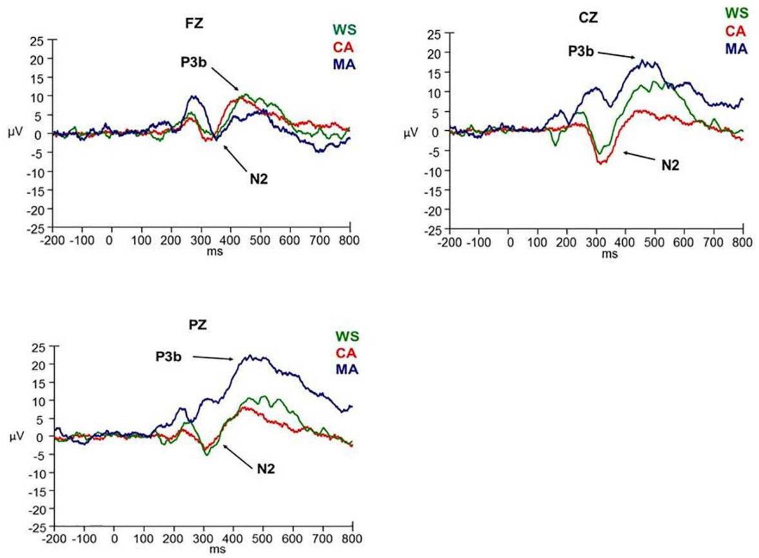

Fig 2. ERP waveforms in response to the target stimulus at FZ, CZ, and PZ electrode sites.

doi:10.1371/journal.pone.0170180.g002

Table 1. Mean peak novel N2 amplitude (μv) and mean peak latency (ms) (SD in parentheses) for the WS, CA, and MA groups at FZ, CZ, & PZ electrode sites.

	Amplitude			Latency		
	WS	CA	MA	WS	CA	MA
FZ	-3.47	-6.28	-8.93	251.05	273.32	260.83
	(2.98)	*(3.23)*	*(6.35)*	*(42.69)*	*(30.67)*	*(42.17)*
CZ	-4.91	-10.79	-2.16	246.26	258.67	256.80
	(6.44)	*(7.19)*	*(7.59)*	*(45.68)*	*(41.49)*	*(48.61)*
PZ	-5.42	-5.95	-3.14	219.31	246.05	221.44
	(9.52)	*(4.64)*	*(9.41)*	*(21.14)*	*(43.62)*	*(17.25)*

doi:10.1371/journal.pone.0170180.t001

N2 amplitude (Novel). The ANOVA revealed no main effects (p>0.05). However, there was a significant site x group interaction, [F (4, 64) = 6.037, p < .001] on N2 amplitude to the novel stimulus. In line with the P3a analysis reported below, a more focused approach was warranted. T-tests identified significantly lower novel peak N2 amplitude at FZ in the WS group compared with both the CA (t(21) = 2.138, p<0.05, *d* = 0.87) and MA (t(20) = – 2.492, p <0.05, *d* = 1.02) groups, but not between the CA/MA groups (t(23) = 1.332, p>0.05). Greater novel peak N2 amplitude in the CA group at CZ approached significance compared with the WS group (t(21) = -2.031, p = .055, *d* = 0.82), and was significantly greater than the MA group (t(23) = -2.920, p<0.01; *d* = 1.22). No novel peak N2 amplitude differences were observed at CZ between the WS/MA groups (t(20) = -.906, p>0.05) and at PZ between the WS, CA, and MA groups (all p>0.05).

In WS group there were no difference in novel peak N2 amplitude between sites (FZ/CZ, CZ/PZ, and FZ/PZ; all p>0.05). Novel peak N2 amplitude in the CA group was significantly greater at CZ compared with FZ (t(12) = 2.762, p<0.05; *d* = 1.60), and with PZ (t(12) = -3.252, p<0.01; *d* = 1.89), but not between FZ/PZ (t(12) = -.285, p>0.05). In contrast, the MA group exhibited the opposite pattern with a significant decrease in novel peak N2 amplitude from FZ to CZ (t(11) = -3.497, p<0.01 *d* = 2.12) and FZ to PZ (t(11) = -2.491, p<0.05; *d* = 1.50), and no difference between CZ/PZ (t(11) = .531, p>0.05).

In summary, the WS group exhibited significantly attenuated peak N2 amplitude at FZ/CZ compared with the control groups.

N2 latency (Novel). The ANOVA revealed no significant main effect of group [F (2, 32) = 1.352, p>0.05], or its interaction with site [F (4, 64) = .504, p>0.05], whereas a significant main effect of site, [F (2, 64) = 12.015, p<0.001], was observed. The MA group demonstrated a significant decrease in peak latency from CZ to PZ (t(11) = 2.821, p<0.05; *d* = 1.70) whereas no differences were observed in the WS (t(9) = 1.645, p>0.05) and the CA groups (t(13) = 1.375, p>0.05). In all groups a significant decrease in peak latency from FZ to PZ was observed (WS, t(9), = 2.318, p<0.05, *d* = 1.55; CA, t(13) = 2.230, p<0.05, *d* = 1.24; MA, t(11) = 3.779, p<0.05, *d* = 2.28).

N2 target. The N2 (target) amplitude and latency data were calculated from the mean of the raw peak amplitude and latency scores in response to the target stimulus. Descriptive statistics for the peak N2 amplitude and peak N2 latency to the target stimulus are presented in Table 2.

N2 amplitude (Target). The mixed ANOVA found no significant main effect of group, [F (2, 32) = p>0.05], a significant main effect of site, (F (2, 64) = 5.382, p<0.01], and a significant site x group interaction, (F (4, 64) = 7.698, p < .001], to target peak N2 amplitude.

Independent t-tests revealed significantly lower target peak N2 amplitude at FZ in the WS group compared with the MA group (t(20) = 2.888, p<0.01, *d* = 1.18) but not the CA group (t(21) = 1.135, p>0.05), and no difference between the CA/MA groups (t(23) = 1.621,

Table 2. Mean peak target N2 amplitude (μv) and peak latency (ms) (SD in parentheses) for the WS, CA, and MA groups at FZ, CZ, & PZ.

	Amplitude			Latency		
	**WS**	**CA**	**MA**	**WS**	**CA**	**MA**
**FZ**	-2.87	-4.79	-7.93	265.55	266.63	246.14
	(2.74)	*(4.76)*	*(4.94)*	*(29.19)*	*(48.43)*	*(52.94)*
**CZ**	-4.49	-8.85	-1.99	279.46	289.05	223.68
	(4.63)	*(5.97)*	*(4.37)*	*(33.02)*	*(46.98)*	*(43.73)*
**PZ**	-4.20	-3.27	-0.26	264.38	260.28	235.40
	(6.41)	*(4.48)*	*(6.68)*	*(43.23)*	*(54.63)*	*(21.27)*

doi:10.1371/journal.pone.0170180.t002

p>0.05). In contrast, the difference in peak amplitude observed in the CA group at CZ approached significance compared with the WS group (t(21) = 1.907, p = 0.07, *d* = 0.78), and was significantly greater than MA group (t(23) = -3.254, p<0.05, *d* = 1.36). There was no target peak N2 amplitude difference between the WS/MA groups at CZ (t(20) = -1.301, p>0.05) and at PZ for all three group comparisons (all p>0.05).

The WS group showed no difference in target peak N2 amplitude between FZ/CZ, CZ/PZ, and FZ/PZ (all p>0.05); whereas the CA group exhibited a significant increase in peak amplitude from FZ to CZ (t(12) = 3.608, p<0.05, *d* = 2.08), a decrease from CZ to PZ (t(12) = -4.638, p = 0.001, *d* = 2.68), and no difference between FZ/PZ (t(12) = -1.387, p>0.05). In contrast, the MA group exhibited a significant decrease in target peak N2 amplitude from both FZ to CZ (t(11) = -3.23, p<0.05, *d* = 1.95) and FZ to PZ (t(11) = -2.491, p<0.05, *d* = 1.50), but not CZ/PZ (t(11) = -.917, p>0.05).

N2 latency (Target). Analyses violated Mauchly's test of sphericity therefore a Greenhouse-Geisser correction has been applied. The mixed ANOVA reported a significant main effect of group, [F (2, 32) = 5.246, p<0.05], no significant main effect of site, [F (1.662, 53.173) = .726, p>0.05], and no significant site x group interaction, [F (3.323, 53.173) = 1.500, p<0.05], on target peak N2 latency.

No difference in target peak N2 latency was observed at FZ and PZ between the WS, CA, and MA groups (all p>0.05). There was no difference in peak latency at CZ (t(21) = -.548, p>0.05) between the WS and CA groups, but this was significantly delayed in the MA group compared with both the WS (t(20) = 3.317, p<0.01, *d* = 1.48) and CA (t(23) = 3.593, p<0.01), *d* = 1.50) groups.

Both the WS and MA groups exhibited no difference in target peak N2 latency between FZ/CZ, CZ/PZ, and FZ/PZ (all p>0.05). The CA group also showed no peak latency differences between FZ/CZ (t(12) = -1.438, p>0.05), and FZ/PZ (t(12) = .298, p>0.05), but demonstrated a significant decrease in peak latency from CZ to PZ (t(12) = 2.269, p<0.05, *d* = 1.31).

In summary, the WS group reported attenuated N2 peak amplitude in response to the target, but no latency delay.

P3a results

The P3a amplitude data were calculated by subtracting the peak amplitude of the frequent stimulus from the peak amplitude of the novel stimulus, thus the P3a amplitude data reported is the mean difference in peak amplitude between these conditions (see [36]). The P3a latency data were calculated from the mean of the raw peak latency scores in response to the novel stimulus. Descriptive statistics for the mean peak P3a amplitude and mean peak P3a latency are reported in Table 3.

P3a amplitude. There was no significant main effect of group on P3a amplitude, [F (2, 32) = .325, p>0.05]; whereas a significant main effect of site, [F (2, 64) = 11.53, p < .001], and a significant site x group interaction, [F (4, 64) = 3.69, p<0.01], were observed. Follow-up comparisons revealed no difference in peak amplitude between FZ and CZ for the WS (t(9) = -1.690, p>0.05) and CA (t(12) = -.923, p>0.05) groups, whereas a significant increase in peak amplitude from FZ to CZ (t(11) = -2.903, p = 0.01, *d* = 1.75) was observed in the MA group. In contrast, significantly greater peak amplitude at CZ compared with PZ (all p<0.001) was observed in both the WS (t(9) = 5.824, p<0.001, *d* = 3.89) and CA (t(12) = 6.590, p<0.001, *d = 3.81*) groups, whereas no peak amplitude difference was observed between CZ and PZ in the MA group (t(11) = 1.372, p>0.05). The CA group's P3a peak amplitude was significantly greater at FZ compared with PZ (t(12) = 3.371, p<0.01, *d* = 1.95), whereas no significant

Table 3. Mean peak P3a amplitude (μv) and peak latency (ms) for P3a (SD in parentheses) for the WS, CA, and MA groups at FZ, CZ, & PZ electrode sites.

	Amplitude			Latency		
	WS	CA	MA	WS	CA	MA
FZ	11.83	13.30	11.31	413.50	388.78	380.63
	(5.31)	*(3.83)*	*(13.10)*	*(16.82)*	*(20.39)*	*(44.30)*
CZ	13.99	14.21	17.52	418.77	396.78	393.4
	(4.75)	*(4.34)*	*(17.37)*	*(18.4)*	*(19.1)*	*(59.03)*
PZ	9.27	9.51	14.85	415.11	408.46	395.72
	(5.29)	*(4.69)*	*(13.13)*	*(57.65)*	*(43.08)*	*(61.25)*

doi:10.1371/journal.pone.0170180.t003

difference in peak amplitude between these sites was found in the WS (t(9) = 1.706, p>0.05) and MA groups (t(11) = -1.629, p>0.05).

P3a latency. The analyses violated Mauchly's test of sphericity; therefore a Greenhouse-Geisser correction has been applied to the P3a latency results. The ANOVA revealed no significant main effects of group [F (2, 32) = 1.615, p>0.05], site [F(1.202, 38.471) = 1.530, p>0.05] or site by group interaction [F (2.404, 38.471) = .343, p>0.05]. However, since the P3a is typically centred on fronto-central locations (confirmed above for WS and CA groups) it was appropriate to consider a more focused analysis. T-tests identified significantly delayed peak P3a latency in the WS group than the CA group at both FZ (t(21) = 3.103, p<0.01, *d* = 1.26) and at CZ (t(21) = 2.781, p<0.05, *d* = 1.13). The WS group's peak latency at FZ was also significantly delayed than observed in the MA group (t(20) = 2.210, p<0.05, *d* = 0.98), but not at CZ (t(20) = 1.303, p>0.05). There was no difference in peak P3a latency between the CA and MA groups at FZ (t(23) = .599, p>0.05) and CZ (t(20) = .196, p>0.05), and no differences between the WS, CA, and MA groups at PZ (all p>0.05). Analyses revealed a significant increase in peak P3a latency by site from FZ to CZ (t(12) = -2.189, p<0.05, *d* = 1.26), and from FZ to PZ (t(11) = -2.186, p<0.05, *d* = 1.32), but not CZ/PZ (t(11) = -1.313, p>0.05) in the CA group. There was no difference in peak P3a latency by site (all p≥0.05) in both the WS and MA groups. In summary, a significant increase in fronto-central (FZ / CZ) latency was observed in the WS group compared to the CA group, which suggests a delay in the neural mechanism engaged in response to the novel stimulus.

P3b results

The P3b amplitude and latency data were calculated as described for the P3a. Descriptive statistics for the mean peak P3b amplitude and mean peak P3b latency are reported in Table 4.

P3b amplitude. Analyses violated Mauchly's test of sphericity therefore a Greenhouse-Geisser correction was applied. The ANOVA identified a significant main effect of group,

Table 4. Mean peak P3b amplitude (μv) and peak latency (ms) (SD in parentheses) for the WS, CA, and MA groups at FZ, CZ, & PZ electrode sites.

	Amplitude			Latency		
	WS	CA	MA	WS	CA	MA
FZ	9.60	9.79	8.01	459.39	429.94	341.85
	(7.29)	*(6.14)*	*(5.23)*	*(78.90)*	*(35.23)*	*(119.49)*
CZ	7.85	4.43	15.89	486.79	459.16	437.47
	(7.43)	*(7.25)*	*(9.77)*	*(47.01)*	*(62.87)*	*(124.82)*
PZ	6.38	6.22	18.36	429.76	420.59	456.10
	(6.24)	*(6.63)*	*(9.69)*	*(82.31)*	*(54.25)*	*(79.87)*

doi:10.1371/journal.pone.0170180.t004

[F (2, 32) = 4.161, $p<0.05$] and a site x group interaction, [F (3.381, 54.095) = 13.886, $p<0.001$], on the P3b amplitude.

Follow up comparisons using t-tests identified significantly greater peak P3b amplitude in the MA group compared with the WS group at both CZ (t(20) = -2.137, $p<0.05$, $d = 0.88$) and PZ (t(20) = -3.364, $p<0.01$, $d = 0.82$), and with the CA group at CZ (t(23) = -3.348, $p<0.01$, $d = 1.40$) and PZ, (t(23) = 3.683, p = 0.001, $d = 1.53$). In addition, the WS group showed no significant difference in peak P3b amplitude between all sites (all $p>0.05$), whereas the CA group showed significantly greater peak P3b amplitude at FZ compared with CZ (t(12) = 4.156, p = 0.001, $d = 2.40$), FZ compared with PZ (t(12) = 3.075. p = .01, $d = 1.78$), and an increase in peak amplitude from CZ to PZ which approached significance (t(12) = -2.006, p = 0.068, $d = 1.15$). For the MA group, a significant increase in peak P3b amplitude from both FZ to CZ (t(11) = -3.589, $p<0.01$, $d = 2.16$) and FZ to PZ (t(11) = -4.061, $p<0.01$, $d = 2.49$) was observed, but no peak amplitude difference between CZ and PZ (t(11) = -1.450, $p>0.05$).

P3b latency. The ANOVA found no main effect of group ($p >0.05$), a significant main effect of site [F (2, 64) = 3.715, $p<0.05$], and a significant site x group interaction, [F (4, 64) = 2.942, $p<0.05$], on peak P3b latency.

T-tests revealed significantly delayed peak P3b latency at FZ in the WS group compared with the MA group (t(20) = 2.677, $p<0.05$, $d = 1.09$) and with the CA group (t(21) = 2.256, $p<0.05$, $d = 0.98$) but not between the CA and MA groups (t(23) = .340, $p>0.05$). There were no group differences in peak P3b latency at CZ and PZ (all $p>0.05$).

Neither the WS nor CA group exhibited any differences in peak P3b latency between sites (FZ/CZ, CZ/PZ, and FZ/PZ; all $p>0.05$). In contrast, the MA group showed an increase in latency from FZ to CZ that approached significance (t(11) = -2.150, p = 0.06, $d = 1.30$), a significant increase from FZ to PZ (t(11) = -2.559, $p<0.05$, $d = 1.54$), but no latency difference between CZ and PZ (t(11) = -.568, $p>0.05$).

Behavioural results

A one-way ANOVA was applied to the reaction time (RT) data to the target stimulus. There was a significant main effect of group, [F (2, 31) = 6.004, $p<0.01$]. Post hoc comparisons revealed the WS group's RT was significantly slower (mean 500.65ms, SD 64.56) to the target compared with the CA group (mean 422.36ms, SD 32.76) ($d = 1.52$; p = 0.01), but not the MA group (mean 490.67ms, SD 59.54) ($p>0.05$). The CA group's RT was also significantly faster than the MA group ($p<0.05$) showing an increase in speed of response with age as would be expected. Speed of processing in the WS group was comparable to their mental age. There was no difference in accuracy in response to the target, with all groups' performance reaching 100% accuracy. Also there was no significant correlation between behavioural RT and target N2 / P3b latency (all $p>0.05$) in all three groups across all sites.

Discussion

The aim of the current study was to investigate the neuro-cognitive mechanisms engaged during the Oddball task in adults with Williams syndrome (WS) as a measure of attentional and inhibitory control. The paradigm is ideally suited to track different aspects of attention and inhibition within one task. By utilising the strengths of ERPs, the data contribute to understanding the EF profile exhibited in the disorder, showing deficits in the early error monitoring processes required for successful inhibition, and a delay in the processing or disengagement of task-irrelevant stimulus. The results tentatively suggest there are atypicalities in relatively earlier and later ERP components in response to the novel stimulus, and dissociation between involuntary and voluntary attentional processing. The main findings were as follows:

compared to the CA group, the WS group reported attenuated peak N2 amplitude in response to the novel and target stimuli, an increase in peak P3a latency in response the novel stimulus, and no peak P3b amplitude or peak N2 / P3b latency differences in response to the target stimulus. Therefore the use of ERP methodology in the current study has added to our understanding of the executive profile exhibited by individuals with WS (e.g. cognitive disinhibition [19, 21, 22] and which may sub-serve their disproportionate attention to social stimuli [9, 14, 69], thus providing a theoretical contribution of the atypicalities in these neural mechanisms.

Consider first the P3a component related to orientation of attention and inhibition. The P3a amplitude was not particularly informative in terms of the WS group comparison with no significant difference in P3a amplitude between the WS and control groups, irrespective of site. However, inspection of the scalp distributions identified specific group differences. Indeed, consistent with previous research, both the WS and CA groups reported larger peak amplitude fronto-centrally in response to the novel task-irrelevant stimulus as expected [36, 70], whereas the MA group's data reported a centro-parietal distribution [71]. It could be argued that there is similar response to the distracting task-irrelevant stimuli across groups but the latency data may give further clues to inhibitory deficits in the WS population seen at the behavioural level, e.g. [9, 22].

The WS group reported an overall delay in P3a peak latency, compared to both the CA and MA groups. The amplitude data may therefore be indicative of similar levels of attention during the 'automatic' shift in focus to the distracting novel stimulus with the P3a latency suggestive of longer and inefficient, inappropriate stimulus evaluation. This finding is consistent with the delayed P3a peak latency reported in younger adults with WS [54], and young-middle aged adults with Fragile X syndrome [62]. To be clear, as the amplitude of the P3a is thought to highlight the extent of involuntary shifts in attention [72], the results indicate that adults with WS group have the same neural responsivity to the novel stimulus as age-matched typically developing controls, but report a delay in the neural mechanisms required to automatically detach from one task and refocus attention on an unexpected event. When applied to their behavioural profile, this suggests that inappropriate behavioural actions are likely linked to similar orientation of attention to irrelevant stimuli in the environment but less ability to disengage (see atypicalities of disengagement, but not engagement, to social information [16, 69, 73]). Indeed, with reports of attention disengagement difficulties in toddlers with WS [74], the current study tentatively suggests that this may be a difficulty that is exhibited across the developmental spectrum, though this needs to be verified with both cross-sectional and longitudinal analyses.

The results from the P3b data also highlighted an unusual neural profile, in both the adults with WS and CA matched group. Overall there were no significant differences in P3b peak amplitude between the WS and CA adults; however the CA group reported a significant frontal maximum, whilst the MA group reported an enhanced centro-parietal P3b distribution as expected [75]. An anterior shift in P3b distribution is observed with increasing age in typically developing older individuals (~70+ years) [76], but has also been reported in middle-age (~49 years) [77]. This shift is thought to reflect an increasing age-associated reliance on frontally controlled executive processes during contextual updating, a process which is more automatic in younger individuals [78], thus explains the frontal maximum observed in the CA group. In contrast the WS group reported no significant differences in P3b peak amplitude across the three midline sites. The absence of any P3b differences between the frontal, central, and parietal electrodes analysed in the current study infers a less efficient voluntary attentional processing system to the task-relevant stimulus; alternatively it could reflect the recruitment of a wider range of cortical regions during voluntary attentional processing to compensate for the known abnormalities in WS such as reduced parietal grey matter density [79] and disproportionate

decrease in parietal volume ([80] also see [81] for a meta-analysis on dorsal / ventral activity during Oddball paradigms in typical development). Combined with the P3b amplitude profile, the lack of any difference in P3b peak latency between the WS and CA groups in the current study suggests that the Oddball paradigm did not place great demands of sustained attention in our WS cohort, unlike the behavioural data from the SART described elsewhere [22] and which incorporated high *Go* / low *No-Go* methodology. (See [74, 82] for discussions on delineating different aspects of attention between syndromes, due to differences in the domains more or less impaired). Thus our data indicate that, under conditions that do not place great demands on voluntary attentional processes, adults with WS are able to achieve the same behavioural result but through slightly different neural mechanisms. This result is also comparable with adults with ADHD [57], but not younger individuals with ASD who reported delayed P3b peak latency [58].

The results from both the novel and target N2 component also contribute in elucidating atypicalities in the WS neural profile during involuntary and voluntary attentional processing. The WS group did not demonstrate any localised novel or target N2 distributions, evidenced by non-significant differences in peak N2 amplitude across all three midline sites in both conditions. Furthermore, relative to both the CA and MA controls, the WS group reported significantly reduced frontal novel peak N2 amplitude; and, compared to the CA group, a reduction in both the novel and target peak N2 amplitude at the central site which approached significance. This contrasts with the limited published research documenting the N2 in WS, which highlighted atypically enhanced N2 negativity in response to both upright and inverted faces [83, 84], and in response to repeated faces and houses [34]. However, it is important to emphasise that WS is often associated with a pro-social drive and a fascination for looking at faces; therefore the results reported by Mills and colleagues [83, 84] may reflect the atypical neural profile that delineates their propensity for prolonged face gazing [69] and not the executive deficits under investigation in the current study.

One theoretical perspective posits that the N2 component in *Go* / *No-Go* paradigms reflects conflict arising from competition between the execution (target) and the inhibition (novel) of a single response [85]. A larger N2 is typically reported frontally and / or centrally when an overt response needs to be withheld, thus motivated by inhibition of a planned response [86], whereas a reduced novel N2 is indicative of an ongoing propensity to respond [43]. This approach is highly pertinent as the numerically greater N2 amplitude at FZ (CA group) and CZ (MA group) in response to the novel indicates appropriate neural responsivity required for successful inhibition in both typically developing control groups. In contrast, the overall attenuated N2 amplitudes observed in the WS group, especially in response to the novel stimulus, demonstrate deficiencies in earlier components that regulate conflict monitoring processes during *Go/No-Go* discrimination.

However, there are certain methodological issues to consider with the Oddball paradigm adopted in the current study. Both the N2 and P3a may habituate on repeated exposure to the same stimulus, and this habituation continues into second and ongoing blocks of presentation [87]. Furthermore, the N2 is not influenced by task difficulty; rather it is sensitive to perceptual deviation from the other stimuli [88]. Thus, it is possible that the comparable P3a peak amplitude profile reported by the WS and the CA groups reflects habituation processes, whilst the attenuated novel and target peak N2 amplitudes in the WS group are indicative of neuronal dysfunction in perceptually discriminating between the novel and target stimuli from the frequent stimulus, despite object perception being a robust trait [89]. Future research adopting an Oddball paradigm would benefit from including unrepeated novel stimuli as this could provide a purer P3a response, and more distinct differences between the novel and target stimuli in order to eradicate these possible confounds. It is worthwhile noting that there is much

discussion and research on the task parameters that influence the P300 responses (see [36] for discussion). It was important in the present study to use a paradigm that has successfully generated the ERP components of interest and thereby allow indices of attention and inhibition to be compared between individuals with WS and those developing typically.

To the best of our knowledge the Oddball methodology adopted here has not been used to date in research with WS individuals. In conclusion, the adults with WS reported a delay in their involuntary attentional processes, most likely due to earlier processing deficits evidenced by the attenuated novel N2 amplitude. Deficits in the monitoring of task-relevant and irrelevant stimuli appear comprised in WS at this earlier stage of processing. Their atypical target N2 and comparable P3b profile, combined with their behavioural performance reaching ceiling level, indicates that they are able to overcome attentional processing deficits in response to the target stimulus when more effortful voluntary processing is required. We argue that the P3a latency in the present study is a key index and indicative of inefficient stimulus evaluation and an atypical delay in their involuntary attentional processes, and perhaps also a marker of poor return to the processing of task-relevant stimuli. Due to the heterogeneity of executive processes and myriad measures of inhibitory control further work is warranted using the finding here as the groundwork. Of course, it is highly likely that these attentional and inhibitory atypicalities underlie aspects of not only the cognitive profile of WS but also the behavioural profile we associate with the disorder.

Author Contributions

Conceptualization: JG LR.

Data curation: JG MM.

Formal analysis: JG LR.

Investigation: JG MM.

Methodology: JG LR.

Project administration: JG.

Resources: JG MM.

Software: JG LR.

Supervision: LR DR CH.

Visualization: JG LR.

Writing – original draft: JG.

Writing – review & editing: JG LR DR CH MM.

References

1. Morris CA, Mervis CB. Williams Syndrome and Related Disorders. Annu Rev Genomics Hum Genet. 2000; 1:461–84. doi: 10.1146/annurev.genom.1.1.461 PMID: 11701637
2. Osborne LR, Mervis CB. Rearrangements of the Williams-Beuren syndrome locus: molecular basis and implications for speech and language development. Expert reviews in molecular medicine. 2007; 9(15):1–16. doi: 10.1017/S146239940700035X PMID: 17565757
3. Meyer-Lindenberg A, Mervis CB, Berman KF. Neural mechanisms in Williams syndrome: a unique window to genetic influences on cognition and behaviour. Nature reviews Neuroscience. 2006; 7(5):380–93. doi: 10.1038/nrn1906 PMID: 16760918

4. Porter M, Coltheart M. Cognitive Heterogeneity in Williams Syndrome. Developmental neuropsychology. 2005; 27(2):275–306. doi: 10.1207/s15326942dn2702_5 PMID: 15753050

5. Searcy YM, Lincoln AJ, Rose FE, Klima ES, Bavar N, Korenberg JR, et al. The relationship between age and IQ in adults with Williams syndrome. American Journal on Mental Retardation. 2004; 109 (3):231–6. doi: 10.1352/0895-8017(2004)109<231:TRBAAI>2.0.CO;2 PMID: 15072519

6. Jarrold C, Baddeley AD, Phillips C. Long-Term Memory for Verbal and Visual Information in Down Syndrome and Williams Syndrome: Performance on the Doors and People Test. Cortex. 2007; 43(2):233–47. PMID: 17405669

7. Vicari S, Bellucci S, Carlesimo GA. Visual and spatial long-term memory: differential pattern of impairments in Williams and Down syndromes. Developmental Medicine & Child Neurology. 2005; 47 (05):305–11.

8. Brock J. Language abilities in Williams syndrome: A critical review. Development and psychopathology. 2007; 19(01):97–127.

9. Little K, Riby DM, Janes E, Clark F, Fleck R, Rodgers J. Heterogeneity of social approach behaviour in Williams syndrome: the role of response inhibition. Research in developmental disabilities. 2013; 34 (3):959–67. doi: 10.1016/j.ridd.2012.11.020 PMID: 23291513

10. Mervis CB, Klein-Tasman BP. Williams syndrome: cognition, personality, and adaptive behavior. Mental retardation and developmental disabilities research reviews. 2000; 6(2):148–58. doi: 10.1002/1098-2779(2000)6:2<148::AID-MRDD10>3.0.CO;2-T PMID: 10899809

11. Riby D, Kirk H, Hanley M, Riby L. Stranger danger awareness in Williams syndrome. Journal of Intellectual Disability Research. 2014; 58(6):572–82. doi: 10.1111/jir.12055 PMID: 23718278

12. Jones W, Bellugi U, Lai Z, Chiles M, Reilly J, Lincoln A, et al. II. Hypersociability in Williams syndrome. Cognitive Neuroscience, Journal of. 2000; 12(Supplement 1):30–46.

13. Meyer-Lindenberg A, Hariri AR, Munoz KE, Mervis CB, Mattay VS, Morris CA, et al. Neural correlates of genetically abnormal social cognition in Williams syndrome. Nature neuroscience. 2005; 8(8):991–3. doi: 10.1038/nn1494 PMID: 16007084

14. Frigerio E, Burt DM, Gagliardi C, Cioffi G, Martelli S, Perrett DI, et al. Is everybody always my friend? Perception of approachability in Williams syndrome. Neuropsychologia. 2006; 44(2):254–9. doi: 10.1016/j.neuropsychologia.2005.05.008 PMID: 16005478

15. Jarvinen-Pasley A, Vines BW, Hill KJ, Yam A, Grichanik M, Mills D, et al. Cross-modal influences of affect across social and non-social domains in individuals with Williams syndrome. Neuropsychologia. 2010; 48(2):456–66. doi: 10.1016/j.neuropsychologia.2009.10.003 PMID: 19822162

16. Riby DM, Jones N, Brown PH, Robinson LJ, Langton SR, Bruce V, et al. Attention to faces in Williams syndrome. Journal of autism and developmental disorders. 2011; 41(9):1228–39. doi: 10.1007/s10803-010-1141-5 PMID: 21125323

17. Costanzo F, Varuzza C, Menghini D, Addona F, Gianesini T, Vicari S. Executive functions in intellectual disabilities: a comparison between Williams syndrome and Down syndrome. Research in developmental disabilities. 2013; 34(5):1770–80. doi: 10.1016/j.ridd.2013.01.024 PMID: 23501586

18. Osorio A, Cruz R, Sampaio A, Garayzabal E, Martinez-Regueiro R, Goncalves OF, et al. How executive functions are related to intelligence in Williams syndrome. Research in developmental disabilities. 2012; 33(4):1169–75. doi: 10.1016/j.ridd.2012.02.003 PMID: 22502842

19. Menghini D, Addona F, Costanzo F, Vicari S. Executive functions in individuals with Williams syndrome. Journal of intellectual disability research: JIDR. 2010; 54(5):418–32. doi: 10.1111/j.1365-2788.2010.01287.x PMID: 20537048

20. Hocking DR, Thomas D, Menant JC, Porter MA, Smith S, Lord SR, et al. The interplay between executive control and motor functioning in Williams syndrome. Developmental science. 2013; 16(3):428–42. doi: 10.1111/desc.12042 PMID: 23587040

21. Porter MA, Coltheart M, Langdon R. The neuropsychological basis of hypersociability in Williams and Down syndrome. Neuropsychologia. 2007; 45(12):2839–49. doi: 10.1016/j.neuropsychologia.2007.05.006 PMID: 17597166

22. Greer J, Riby DM, Hamiliton C, Riby LM. Attentional lapse and inhibition control in adults with Williams Syndrome. Research in developmental disabilities. 2013; 34(11):4170–7. doi: 10.1016/j.ridd.2013.08.041 PMID: 24076981

23. Atkinson J, Braddick O. From genes to brain development to phenotypic behavior:" dorsal-stream vulnerability" in relation to spatial cognition, attention, and planning of actions in Williams syndrome (WS) and other developmental disorders. Progress in brain research. 2010; 189:261–83.

24. Scerif G, Cornish K, Wilding J, Driver J, Karmiloff-Smith A. Visual search in typically developing toddlers and toddlers with Fragile X or Williams syndrome. Developmental science. 2004; 7(1):116–30. PMID: 15323123

25. Rhodes SM, Riby DM, Park J, Fraser E, Campbell LE. Executive neuropsychological functioning in individuals with Williams syndrome. Neuropsychologia. 2010; 48(5):1216–26. doi: 10.1016/j.neuropsychologia.2009.12.021 PMID: 20026085

26. Simmonds DJ, Pekar JJ, Mostofsky SH. Meta-analysis of Go/No-go tasks demonstrating that fMRI activation associated with response inhibition is task-dependent. Neuropsychologia. 2008; 46(1):224–32. doi: 10.1016/j.neuropsychologia.2007.07.015 PMID: 17850833

27. Mobbs D, Eckert MA, Mills D, Korenberg J, Bellugi U, Galaburda AM, et al. Frontostriatal dysfunction during response inhibition in Williams syndrome. Biological psychiatry. 2007; 62(3):256–61. doi: 10.1016/j.biopsych.2006.05.041 PMID: 16996488

28. Rhodes SM, Riby DM, Matthews K, Coghill DR. Attention-deficit/hyperactivity disorder and Williams syndrome: Shared behavioral and neuropsychological profiles. Journal of clinical and experimental neuropsychology. 2011; 33(1):147–56. doi: 10.1080/13803395.2010.495057 PMID: 20700845

29. Robertson IH, Manly T, Andrade J, Baddeley BT, Yiend J. Oops!': performance correlates of everyday attentional failures in traumatic brain injured and normal subjects. Neuropsychologia. 1997; 35(6):747–58. PMID: 9204482

30. Smith AD, Gilchrist ID, Hood B, Tassabehji M, Karmiloff-Smith A. Inefficient search of large-scale space in Williams syndrome: further insights on the role of LIMK1 deletion in deficits of spatial cognition. Perception. 2009; 38(5):694–701. PMID: 19662944

31. Jones W, Bellugi U, Lai Z, Chiles M, Reilly J, Lincoln A, et al. II. Hypersociability in Williams syndrome. Journal of Cognitive Neuroscience. 2000; 12(Supplement 1):30–46.

32. Sergeant J. The cognitive-energetic model: an empirical approach to attention-deficit hyperactivity disorder. Neuroscience & Biobehavioral Reviews. 2000; 24(1):7–12.

33. Mills DL, Alvarez TD, St George M, Applebaum LG III. Electrophysiological Studies of face processing in adults with Williams syndrome. Journal of Cognitive Neuroscience. 2000; 12(Suplement1); 47–67.

34. Key AP, Dykens EM. Face repetition detection and social interest: An ERP study in adults with and without Williams syndrome. Social neuroscience. 2015.

35. Donchin E, Ritter W, McCallum WC. Cognitive psychophysiology: The endogenous components of the ERP. Event-related brain potentials in man. 1978:349–411.

36. Polich J. Updating P300: an integrative theory of P3a and P3b. Clinical neurophysiology: official journal of the International Federation of Clinical Neurophysiology. 2007; 118(10):2128–48.

37. Bachiller A, Romero S, Molina V, Alonso JF, Mañanas MA, Poza J, et al. Auditory P3a and P3b neural generators in schizophrenia: An adaptive sLORETA P300 localization approach. Schizophrenia research. 2015; 169(1):318–25.

38. Smith JL, Johnstone SJ, Barry RJ. Movement-related potentials in the Go/NoGo task: The P3 reflects both cognitive and motor inhibition. Clinical neurophysiology. 2008; 119(3):704–14. doi: 10.1016/j.clinph.2007.11.042 PMID: 18164657

39. Goldstein A, Spencer KM, Donchin E. The influence of stimulus deviance and novelty on the P300 and novelty P3. Psychophysiology. 2002; 39(06):781–90.

40. Barron E, Riby LM, Greer J, Smallwood J. Absorbed in thought the effect of mind wandering on the processing of relevant and irrelevant events. Psychological science. 2011.

41. Riby LM, Orme E. A familiar pattern? Semantic memory contributes to the enhancement of visuo-spatial memories. Brain and cognition. 2013; 81(2):215–22. doi: 10.1016/j.bandc.2012.10.011 PMID: 23262176

42. Daffner KR, Alperin BR, Mott KK, Tusch E, Holcomb PJ. Age-related differences in early novelty processing: Using PCA to parse the overlapping anterior P2 and N2 components. Biological psychology. 2015.

43. Folstein JR, Van Petten C. Influence of cognitive control and mismatch on the N2 component of the ERP: a review. Psychophysiology. 2008; 45(1):152–70. doi: 10.1111/j.1469-8986.2007.00602.x PMID: 17850238

44. Picton TW, Alain C, Otten L, Ritter W, Achim A, eacute. Mismatch negativity: different water in the same river. Audiology and Neurotology. 2000; 5(3–4):111–39 PMID: 10859408

45. Polich J. Theoretical overview of P3a and P3b: Springer; 2003.

46. Kähkönen S, Ahveninen J, Pekkonen E, Kaakkola S, Huttunen J, Ilmoniemi RJ, et al. Dopamine modulates involuntary attention shifting and reorienting: an electromagnetic study. Clinical Neurophysiology. 2002; 113(12):1894–902. PMID: 12464326

47. Riby LM. The joys of spring. Experimental psychology. 2013.

48. Conroy MA, Polich J. Normative variation of P3a and P3b from a large sample: Gender, topography, and response time. Journal of Psychophysiology. 2007; 21(1):22–32.

49. del Re EC, Spencer KM, Oribe N, Mesholam-Gately RI, Goldstein J, Shenton ME, et al. Clinical high risk and first episode schizophrenia: auditory event-related potentials. Psychiatry Research: Neuroimaging. 2015; 231(2):126–33. doi: 10.1016/j.pscychresns.2014.11.012 PMID: 25557063

50. Osborne A, Riby LM. The Effect of Predisposing Risk Factors of an Eating Disorder on Response Inhibition and Working Memory: An Event-Related Potentials Study. Research in Psychology and Behavioral Sciences. 2016; 4(1):1–6.

51. Cléry H, Bonnet-Brilhault F, Lenoir P, Barthelemy C, Bruneau N, Gomot M. Atypical visual change processing in children with autism: an electrophysiological study. Psychophysiology. 2013; 50(3):240–52. doi: 10.1111/psyp.12006 PMID: 23316882

52. Grice S, Spratling MW, Karmiloff-Smith A, Halit H, Csibra G, de Haan M, et al. Disordered visual processing and oscillatory brain activity in autism and Williams Syndrome. Neuroreport. 2001; 12 (12):2697–700. PMID: 11522950

53. Haas BW, Mills D, Yam A, Hoeft F, Bellugi U, Reiss A. Genetic influences on sociability: heightened amygdala reactivity and event-related responses to positive social stimuli in Williams syndrome. The Journal of neuroscience: the official journal of the Society for Neuroscience. 2009; 29(4):1132–9.

54. Key AP, Dykens EM. Electrophysiological study of local/global processing in Williams syndrome. Journal of neurodevelopmental disorders. 2011; 3(1):28–38. doi: 10.1007/s11689-010-9064-1 PMID: 21484595

55. Segalowitz SJ, Davies PL. Charting the maturation of the frontal lobe: An electrophysiological strategy. Brain and cognition. 2004; 55(1):116–33. doi: 10.1016/S0278-2626(03)00283-5 PMID: 15134847

56. Stige S, Fjell AM, Smith L, Lindgren M, Walhovd KB. The development of visual P3a and P3b. Developmental neuropsychology. 2007; 32(1):563–84. doi: 10.1080/87565640701361096 PMID: 17650994

57. Barry RJ, Clarke AR, McCarthy R, Selikowitz M, Brown CR, Heaven PC. Event-related potentials in adults with Attention-Deficit/Hyperactivity Disorder: an investigation using an inter-modal auditory/visual oddball task. International Journal of Psychophysiology. 2009; 71(2):124–31. doi: 10.1016/j.ijpsycho.2008.09.009 PMID: 19022305

58. Sokhadze E, Baruth J, Tasman A, Sears L, Mathai G, El-Baz A, et al. Event-related potential study of novelty processing abnormalities in autism. Applied Psychophysiology and Biofeedback. 2009; 34 (1):37–51. doi: 10.1007/s10484-009-9074-5 PMID: 19199028

59. Karmiloff-Smith A. Challenging the use of adult neuropsychological models for explaining neurodevelopmental disorders: Develop ed versus develop ing brains. The Quarterly Journal of Experimental Psychology. 2013; 66(1):1–14. doi: 10.1080/17470218.2012.744424 PMID: 23173948

60. Dunn L, Dunn L, Whetton C, Burley J. The British Picture Vocabulary Scale, 2nd edn (Windsor: NFER-Nelson). 1997.

61. Oldfield RC. The assessment and analysis of handedness: the Edinburgh inventory. Neuropsychologia. 1971; 9(1):97–113. PMID: 5146491

62. Van der Molen M, Van der Molen M, Ridderinkhof K, Hamel B, Curfs L, Ramakers G. Auditory and visual cortical activity during selective attention in fragile X syndrome: a cascade of processing deficiencies. Clinical Neurophysiology. 2012; 123(4):720–9. doi: 10.1016/j.clinph.2011.08.023 PMID: 21958658

63. Brinkman MJ, Stauder JE. Development and gender in the P50 paradigm. Clinical Neurophysiology. 2007; 118(7):1517–24. doi: 10.1016/j.clinph.2007.04.002 PMID: 17509936

64. Klem GH, Lüders HO, Jasper H, Elger C. The ten-twenty electrode system of the International Federation. Electroencephalogr Clin Neurophysiol. 1999; 52(suppl.):3.

65. St-Louis-Deschênes M, Moore R, Ellemberg D. The Selective Effect of Acute Aerobic Exercise on Neuroelectric Indices of Attention during Development. Pediat Therapeut. 2015; 5(238):2161–0665.1000238.

66. Steiner GZ, Brennan ML, Gonsalvez CJ, Barry RJ. Comparing P300 modulations: Target-to-target interval versus infrequent nontarget-to-nontarget interval in a three-stimulus task. Psychophysiology. 2013; 50(2):187–94. doi: 10.1111/j.1469-8986.2012.01491.x PMID: 23153378

67. Johnson R. On the neural generators of the P300 component of the event-related potential. Psychophysiology. 1993; 30:90-. PMID: 8416066

68. Hedges L, Olkin I. Statistical models for meta-analysis. New York: Academic Press Hedges, LV, & Pigott, TD (2001) The power of statistical tests in meta-analysis Psychological Methods. 1985; 6:203–17.

69. Riby DM, Hancock PJ. Viewing it differently: social scene perception in Williams syndrome and autism. Neuropsychologia. 2008; 46(11):2855–60. doi: 10.1016/j.neuropsychologia.2008.05.003 PMID: 18561959

70. Wetzel N, Schröger E, Widmann A. The dissociation between the P3a event-related potential and behavioral distraction. Psychophysiology. 2013; 50(9):920–30. doi: 10.1111/psyp.12072 PMID: 23763292
71. Comerchero MD, Polich J. P3a and P3b from typical auditory and visual stimuli. Clinical Neurophysiology. 1999; 110(1):24–30. PMID: 10348317
72. Escera C, Yago E, Alho K. Electrical responses reveal the temporal dynamics of brain events during involuntary attention switching. European Journal of Neuroscience. 2001; 14(5):877–83. PMID: 11576192
73. Lincoln A, Lai Z, Jones W. Shifting attention and joint attention dissociation in Williams syndrome: implications for the cerebellum and social deficits in autism. Neurocase. 2002; 8(2):226–32.
74. Brown JH, Johnson MH, Paterson SJ, Gilmore R, Longhi E, Karmiloff-Smith A. Spatial representation and attention in toddlers with Williams syndrome and Down syndrome. Neuropsychologia. 2003; 41 (8):1037–46. PMID: 12667539
75. Thomas KM, Nelson CA. Age-related changes in the electrophysiological response to visual stimulus novelty: A topographical approach. Electroencephalography and clinical Neurophysiology. 1996; 98 (4):294–308. PMID: 8641151
76. Kopp B, Lange F, Howe J, Wessel K. Age-related changes in neural recruitment for cognitive control. Brain and cognition. 2014; 85:209–19. doi: 10.1016/j.bandc.2013.12.008 PMID: 24434022
77. Smit DJ, Posthuma D, Boomsma DI, de Geus EJ. Genetic contribution to the P3 in young and middle-aged adults. Twin Research and Human Genetics. 2007; 10(02):335–47.
78. Velanova K, Lustig C, Jacoby LL, Buckner RL. Evidence for frontally mediated controlled processing differences in older adults. Cerebral cortex. 2007; 17(5):1033–46. doi: 10.1093/cercor/bhl013 PMID: 16774962
79. Reiss AL, Eckert MA, Rose FE, Karchemskiy A, Kesler S, Chang M, et al. An experiment of nature: brain anatomy parallels cognition and behavior in Williams syndrome. The Journal of neuroscience: the official journal of the Society for Neuroscience. 2004; 24(21):5009–15.
80. Chiang M-C, Reiss AL, Lee AD, Bellugi U, Galaburda AM, Korenberg JR, et al. 3D pattern of brain abnormalities in Williams syndrome visualized using tensor-based morphometry. NeuroImage. 2007; 36(4):1096–109. doi: 10.1016/j.neuroimage.2007.04.024 PMID: 17512756
81. Kim H. Involvement of the dorsal and ventral attention networks in oddball stimulus processing: a meta-analysis. Human brain mapping. 2014; 35(5):2265–84. doi: 10.1002/hbm.22326 PMID: 23900833
82. Cornish K, Scerif G, Karmiloff-Smith A. Tracing syndrome-specific trajectories of attention across the lifespan. Cortex. 2007; 43(6):672–85. PMID: 17710820
83. Mills DL, Alvarez TD, George MS, Appelbaum LG, Bellugi U, Neville H. Linking Cognitive Neuroscience and Molecular Genetics: New Perspectives from Williams Syndrome-III. Electrophysiological Studies of Face Processing in Williams Syndrome. Journal of Cognitive Neuroscience. 2000; 12(1):47–64.
84. Mills DL, Dai L, Fishman I, Yam A, Appelbaum LG, St George M, et al. Genetic mapping of brain plasticity across development in Williams syndrome: ERP markers of face and language processing. Developmental neuropsychology. 2013; 38(8):613–42. doi: 10.1080/87565641.2013.825617 PMID: 24219698
85. Nieuwenhuis S, Yeung N, Van Den Wildenberg W, Ridderinkhof KR. Electrophysiological correlates of anterior cingulate function in a go/no-go task: effects of response conflict and trial type frequency. Cognitive, affective, & behavioral neuroscience. 2003; 3(1):17–26.
86. Bruin K, Wijers A. Inhibition, response mode, and stimulus probability: a comparative event-related potential study. Clinical Neurophysiology. 2002; 113(7):1172–82. PMID: 12088714
87. Courchesne E, Hillyard SA, Galambos R. Stimulus novelty, task relevance and the visual evoked potential in man. Electroencephalography and clinical neurophysiology. 1975; 39(2):131–43. PMID: 50210
88. Polich J, Comerchero MD. P3a from visual stimuli: typicality, task, and topography. Brain topography. 2003; 15(3):141–52. PMID: 12705810
89. Landau B, Hoffman JE, Kurz N. Object recognition with severe spatial deficits in Williams syndrome: sparing and breakdown. Cognition. 2006; 100(3):483–510. doi: 10.1016/j.cognition.2005.06.005 PMID: 16185678
